# Case report: Anti septin-5-encephalitis as a treatable cause of cerebellar ataxia and psychiatric symptoms

**DOI:** 10.3389/fneur.2023.1220295

**Published:** 2023-06-26

**Authors:** Johannes Wischmann, Kathrin Borowski, Joachim Havla, Franziska S. Thaler, Tobias Winkler, Tobias Jung, Andreas Straube, Ilias Masouris

**Affiliations:** ^1^Department of Neurology, University Hospital, Ludwig-Maximilians-University, Munich, Germany; ^2^Clinical Immunological Laboratory Prof. Dr. med. Winfried Stöcker, Luebeck, Germany; ^3^Institute of Clinical Neuroimmunology, University Hospital, Ludwig-Maximilians-Universität Munich, Munich, Germany; ^4^Biomedical Center, Medical Faculty, Ludwig-Maximilians-Universität Munich, Munich, Germany; ^5^Munich Cluster for Systems Neurology (SyNergy), Munich, Germany; ^6^Department of Neurology, kbo-Inn-Salzach-Klinikum, Wasserburg am Inn, Germany

**Keywords:** case report, septin-5, autoimmune encephalitis, bortezomib, cerebellar syndrome

## Abstract

**Objectives:**

Anti-septin-5 encephalitis is a rare disease with only few published cases, mainly based on retrospective CSF and serum analyses. Predominant symptoms are cerebellar ataxia and oculomotor abnormalities. Due to the rareness of the disease, treatment recommendations are scarce. Herein, we prospectively describe the clinical course of a female patient with anti-septin-5 encephalitis.

**Methods:**

We describe diagnostic workup, treatment and follow-up of a 54-year-old patient presenting with vertigo, unsteady gait, lack of drive and behavioral changes.

**Results:**

Clinical examination revealed severe cerebellar ataxia, saccadic smooth pursuit, upbeat-nystagmus, and dysarthria. Additionally, the patient presented with a depressive syndrome. MRI of the brain and spinal cord were normal. CSF analysis showed lymphocytic pleocytosis (11 cells/μl). Extensive antibody testing revealed anti septin-5 IgG in both CSF and serum without coexisting anti-neuronal antibodies. PET/CT detected no signs of malignancy. Corticosteroids, plasma exchange, and rituximab led to transient clinical improvement followed by relapse. Re-applied treatment with plasma exchange followed by bortezomib resulted in moderate but sustained clinical improvement.

**Discussion:**

Anti septin-5 encephalitis represents a rare but treatable and therefore relevant differential diagnosis in patients with cerebellar ataxia. Psychiatric symptoms can be observed in anti septin-5 encephalitis. Immunosuppressive treatment including bortezomib is moderately effective.

## Introduction

Anti-septin-5 encephalitis is an extremely rare antibody-associated autoimmune disorder, with only few reported cases so far. Most cases have been retrospectively diagnosed from archived sera and cerebrospinal fluid (CSF) of patients with initial diagnosis of encephalitis of unknown origin (1–3). The clinical manifestations of the disease can vary, but progressive cerebellar ataxia and ocular movement abnormalities are consistently reported, with onset at a median age of 59 years (4). In some cases, psychiatric symptoms were reported in patients with septin-5-IgG and coexisting septin-7-IgG (3). MRI and CSF findings were heterogeneous in reported cases varying from normal CSF findings to elevated leukocyte count and/or protein levels. Response to immunotherapy was variable in reported cases with a rather limited overall benefit; however, spontaneous remission was also observed (1, 2). The detailed pathomechanism behind autoimmune septin-IgG related encephalitis remains unclear.

## Case report

**Figure 1:**
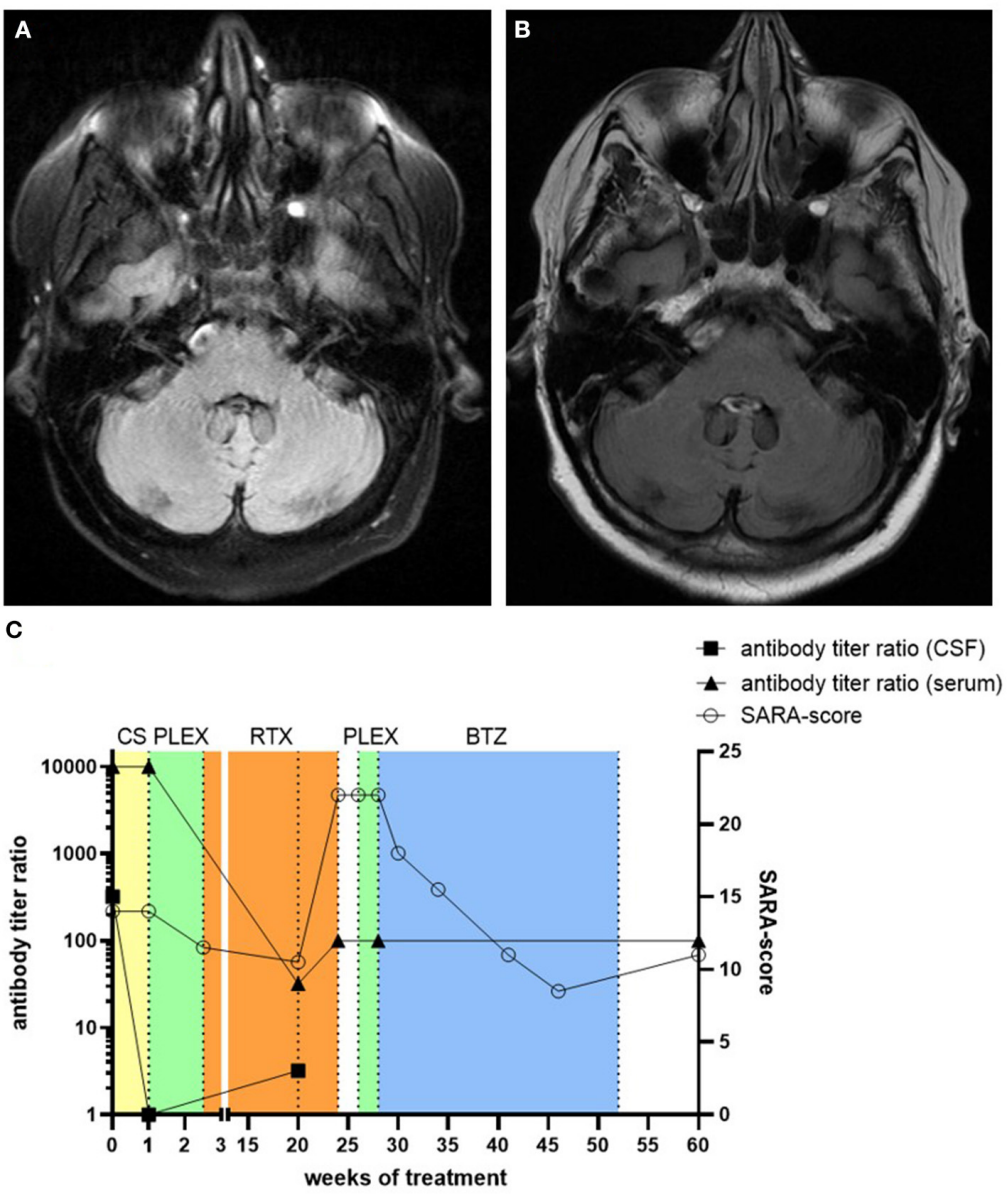
Native axial FLAIR MR-images of the patient 4 months after symptom onset **(A)** and after clinical deterioration 5 months after treatment with rituximab **(B)**. No cerebellar atrophy or inflammatory lesions were detected. The clinical course of the patient at different time-points with corresponding treatment regimens, SARA-score values, and antibody titer ratio in both CSF and serum is depicted **(C)**. CS, corticosteroids; RTX, rituximab; PLEX, plasma exchange; BTZ, bortezomib.

**Figure 2:**
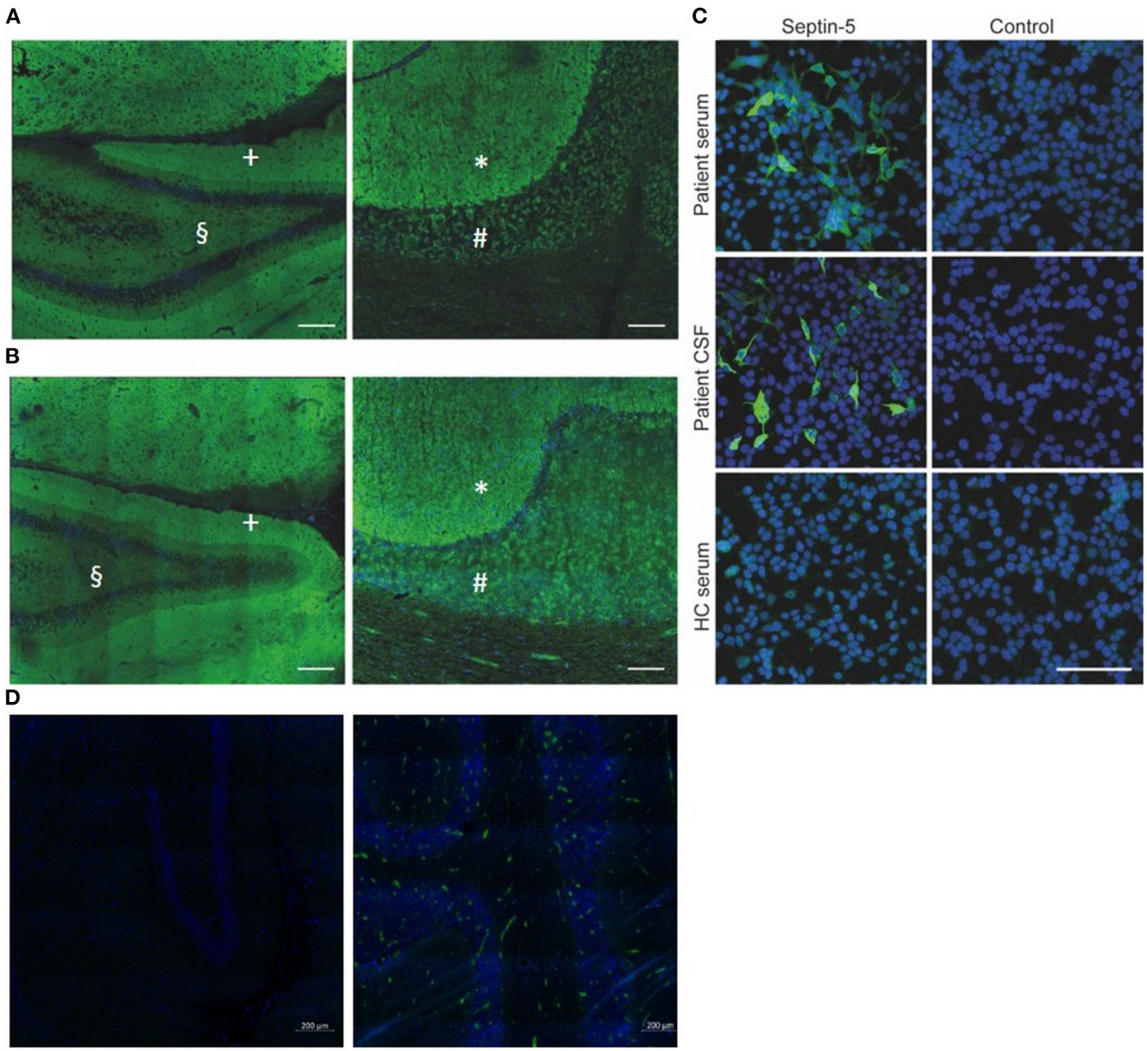
**(A, B)** Strong neuropil staining of rat hippocampus (**A** left, **B** left; scale bar: 200 μm) and primate cerebellum (**A** right, **B** right; scale bar: 100 μm) after incubation with patient CSF **(A)** and serum **(B)**, both from the time of diagnosis. The inner molecular layer (§) of the hippocampus exhibits a weaker immune-reactivity, compared to the outer layer (+). The molecular layer of the cerebellum (*) exhibits a stronger immune-reactivity as compared to the granular layer (#). **(C)** Cell-based assay with human Septin-5 transfected HEK-293 T cells confirms the presence of antibodies against Septin-5 in both patient CSF and serum (dilutions: Serum 1:100, CSF 1:10; scale bar: 100 μm). **(D)** Negative controls of rat hippocampus **(left)** and primate cerebellum **(right)**. Weak immune-reactivity is due to secondary antibodies within the vessels. Green: Alexa-488 Anti-human IgG, blue: DAPI. CSF, cerebrospinal fluid; HC, healthy control.

A 54-year-old woman presented to our hospital with progressive vertigo, oscillopsia, and severe gait and limb ataxia developing over 3 months. She also experienced behavioral changes and severe depressive symptoms, including a lack of drive and passive suicidal thoughts, several months before the onset of her cerebellar symptoms. Her medical history included arterial hypertension, rheumatoid arthritis, and Graves' disease. She had no pertinent family history, and there was no record of alcohol or drug abuse in her personal history. At the time of admission, she was on treatment with antihypertensive drugs, L-thyroxine, quetiapine, and sertraline. On neurological examination, she presented severe left-sided cerebellar limb as well as trunk ataxia, dysarthria, upbeat nystagmus, and a saccadic smooth pursuit. Her initial Scale for the assessment and rating of ataxia (SARA) score was 14 (range 0–40; higher values indicate more severe ataxia). Extended laboratory blood examinations yielded no pathological results. Magnetic resonance imaging (MRI) of the brain and spinal cord showed no abnormalities (Figure 1A). Cerebrospinal fluid (CSF) analysis revealed a lymphocytic pleocytosis (11 cells/μl; reference-value: ≤ 5/μl), with normal protein, normal CSF/serum-quotient of albumin and without CSF- and/or serum-specific oligoclonal bands. Extensive testing of anti-neuronal antibodies, assessed by BIOCHIP mosaic cell-based immunofluorescence assays (Supplementary Table 1), revealed serum (1:10.000; reference value: < 1:100) and CSF (1:320; reference-value: negative) anti-septin-5 IgG (Figure 2); no other auto-antibodies were detected. Tests for thyroid function and antibodies, including TSH (Thyroid-Stimulating Hormone), T_3_ (Triiodothyronine), T_4_ (thyroxine), auto-antibodies against TPO (Thyroid peroxidase), thyroglobulin and TSH-receptor, were normal. Whole-body FDG-positron emission tomography/computed tomography (PET/CT) scan without dedicated brain imaging showed no signs of malignancy.

The patient was initially treated with intravenous methylprednisolone over 5 days (1 g per day), followed by oral tapering starting with 80 mg prednisone per day, but this treatment did not produce any significant clinical effect (Figure 1C). Subsequent CSF analysis during prednisone taper showed no signs of inflammation anymore. Furthermore, anti-septin-5 antibodies decreased in the CSF (1:1), while serum levels remained high (1:10.000). Subsequently, the patient received seven cycles of plasma exchange, which led to moderate clinical improvement, with her SARA score decreasing to 11.5 points. Plasma exchange was followed by B-cell depleting therapy with the monoclonal anti-CD20-antibody rituximab (2 × 1 g with an interval of 2 weeks). After 5 months, the patient showed clinical improvement, with a SARA score of 10.5 points. Psychiatric symptoms also showed significant remission, and she was able to discontinue medication with quetiapine and sertraline.

Although B cells remained undetectable in serum and anti-septin IgG remained low both in CSF (1:3.2) and serum (1:32), 5 months after initiation of rituximab treatment the patient was re-admitted to our hospital with a severe deterioration of both her neurological and psychiatric symptoms. Her SARA score had increased to 22, and we also found increased anti-septin IgG titer in serum (1:100), while MRI still showed no signs of inflammation or degeneration (Figure 1B). No lumbar puncture was performed at this or any further timepoints. Five cycles of plasma exchange was ineffective this time. Therefore, we initiated treatment with the proteasome inhibitor bortezomib (1.3 mg/m^2^ body surface per cycle). Three cycles were applied over 5 months without any adverse effects, which led to an overall moderate improvement of both her neurological and psychiatric symptoms. In a follow-up examination 2 months after the last cycle of bortezomib, her SARA score was 11, but the anti-septin IgG titer in serum remained at 1:100.

## Discussion

Anti-septin-5 encephalitis is a newly described disease with a limited number of reported cases. In this study, we present a prospectively diagnosed and monitored case of anti-septin-5 cerebellitis with distinctive symptoms, including behavioral changes and a severe depressive syndrome. Our patient's treatment with steroids, plasma exchange, rituximab, and bortezomib resulted in a moderate clinical effect of both her neurological and psychiatric symptoms.

In line with previously reported cases, our patient displayed progressive cerebellar ataxia and oculomotor abnormalities. However, we observed additional behavioral changes and a severe depressive syndrome, which has been described before in association with coexisting septin-7 IgG but not with septin-5 (3). We did not detect any other anti-neuronal antibodies, such as anti-glutamic acid decarboxylase (GAD) or N-type calcium channel antibody (CCN), which were described in other cases (1). Cognitive and behavioral alterations have already been described as being associated with cerebellar lesions, termed as Cerebellar Cognitive and Affective Syndrome (CCAS) (5). Although a secondary adjustment disorder cannot be excluded, the rapid reduction in psycho-behavioral symptoms following immunotherapy supports a causal link to septin-5 IgG.

Although detailed pathophysiology of anti septin-5 IgG is unclear, septines play a crucial role in the CNS, organizing neuronal cytoskeletal development and regulating endo- and exocytosis at synaptic terminals, so it is feasible that disrupting their function may lead to neurological deficits (6–8). Apart from septin-5, septin-3 and septin-7 are also associated with autoimmune CNS diseases (3, 9). Whether there is direct pathogenic effect of anti-septin-5 IgG or other components such as T-cell mediated cytotoxicity also play a role is unclear. However, there are hints pointing toward a direct pathogenicity, although this needs further confirmation (3).

Similar to most published cases, repeated MRI-scans showed no evidence of neuroinflammation, a disturbed blood-brain barrier, or atrophy. In line with previously reported cases, initial CSF analysis revealed a lymphocytic pleocytosis (1). Subsequent lumbar puncture after treatment with intravenous methylprednisone and oral prednisone taper yielded normal results, which may be interpreted as a paraclinical response to cortisone. The detected anti-septin-5 IgG titers in serum (1:10.000) was comparable to previously described serologic findings and antibody titers (1). We observed no evidence of malignancy in our patient as was the case in the other published cases. However, we did not perform any follow-up screening for malignancies. Still, the number of described cases is still too low to exclude a paraneoplastic etiology of anti-septin-5 encephalitis (10).

In our patient, corticosteroids, plasma exchange, and rituximab showed a significant therapeutic effect, as it was described in previous studies, which however was only temporary. Unfortunately, the patient remained clinically stable for only 5 months, before relapsing with a more severe syndrome than initially. One prodromal indication of the clinical deterioration could be the slight increase in CSF anti-septin-5 IgG titers (1:1 → 1:3.2) 1 month before. However, no further lumbar punctures were performed subsequently to support this. Since anti-septin-antibodies remained detectable in serum and CSF at the time of clinical deterioration despite ongoing B-cell depletion, we opted for a treatment with the proteasome inhibitor bortezomib, which is known to promote apoptosis of plasma cells that cannot be depleted with rituximab (11). Bortezomib led to a moderate but sustained improvement of the neurological symptoms for 8 months up to now. The use of bortezomib, which has been described before in refractory autoimmune encephalitis, especially in N-methyl-d-aspartate receptor (NMDAR) encephalitis, may be an option in autoimmune septin-5 encephalitis (12).

In conclusion, anti-septin-5 cerebellar ataxia should be considered as differential diagnosis in patients presenting with a global cerebellar syndrome. Psychiatric symptoms represent a possible additional manifestation of anti-septin-5 encephalitis. Immunotherapy including plasma exchange, rituximab, and bortezomib are potentially effective treatment options. However, further studies with larger cohorts are required to provide specific therapeutic recommendations.

## Data availability statement

The raw data supporting the conclusions of this article will be made available by the authors, without undue reservation.

## Ethics statement

Ethical review and approval was not required for the study on human participants in accordance with the local legislation and institutional requirements. The patients/participants provided their written informed consent to participate in this study. Written informed consent was obtained from the individual(s) for the publication of any potentially identifiable images or data included in this article.

## Author contributions

JW and IM designed the study, collected the clinical data, and wrote the first draft of the manuscript. JW, IM, JH, FT, TW, TJ, and AS treated the patient. KB provided the histopathological images. JW, IM, JH, FT, and AS interpreted the clinical data. JH, FT, and AS co-wrote the manuscript. All authors discussed the results reviewed and commented the manuscript. All authors contributed to the article and approved the submitted version.
