# Supplementary material for: Case report: Anti septin-5-encephalitis as a treatable cause of cerebellar ataxia and psychiatric symptoms
Source: Front Neurol. 2023 Jun 26;14:1220295. doi: 10.3389/fneur.2023.1220295 (PMC10331165; doi:10.3389/fneur.2023.1220295)
Supplement: Supplementary file 1 [file Table_1.DOCX]

Supplemental table 1

Supplemental table 1: Tested antineuronal antibodies through BIOCHIP mosaic cell-based immunofluorescence assays. Rat hippocampus and both rat and primate cerebellum was incubated with patient serum and cerebrospinal fluid.

| **Serum** | **Cerebrospinal fluid (CSF)** |
| --- | --- |
| Septin- 1-7, Septin-complex, Hu, Ri, ANNA-3, Yo, Tr/DNER, Ma/Ta, GAD65, Amphiphysin, Aquaporin-4, MOG, NMDA-receptor, AMPA-receptor, GABA-b-receptors, LGI1, CASPR2, IgLON5, ZIC4, DPPX, anti-myelin, anti-GARPVIII, Glycin-receptors, mGluR1, mGluR5, GABA-a-receptors, Rho GTPase activating protein 26, Recoverin, GluR02, Flotillin-1/2, TPR1, Homer 3, Neurochondrin, Neurexin-3-alpha, ERC1, Sez612, AP3B3, Contactin 1, Neurofascin 155, Neurofascin 166, AT1A3, KCNA2 and Dopamine-receptors 2 | Septin- 1-7, Septin-complex, Aquaporin-4, NMDA-receptors, GABA-b-receptors, CASPR2, Hu, Ri, ANNA-3, Yo, Tr/DNER, Myelin, Ma/Ta, GAD65, Amphiphysin, AMPA-receptor, LGI1, ZIC4, DPPX, CARPVIII, Glycin-receptor, mGluR1, mGluR5, GABA-a-receptors, Rho GTPase activating protein 26, ITPR1, Homer 3, MOG, Recoverin, Neurochondrin, GluRD2, Flotillin-1/2, IgLON5, Neurexin-3-alpha, ERC1, Sez612, AP3B2, Contactin 1, Neurofascin 155, Neurofascin 186, AT1A3, KCNA2 and Dopamine-receptors 2 |
